# The effect of rooming-in on duration of breastfeeding: A systematic review of randomised and non-randomised prospective controlled studies

**DOI:** 10.1371/journal.pone.0215869

**Published:** 2019-04-25

**Authors:** Chin Ang Ng, Jacqueline J. Ho, Zcho Huey Lee

**Affiliations:** 1 C/O Department of Paediatrics, RSCI & UCD Malaysia Campus, George Town, Penang, Malaysia; 2 Department of Paediatrics, RSCI & UCD Malaysia Campus, George Town, Penang, Malaysia; University of Ghana, GHANA

## Abstract

**Background:**

The benefits of six months exclusive breastfeeding are well established for both mother and infant. One of the 10 steps of the Baby Friendly Hospital Initiative is rooming-in (mother and baby together in the same room throughout hospitalisation). A Cochrane review found only one randomised controlled trial (RCT) examining the effects of continuous rooming-in versus nursery care on breastfeeding duration, and concluded there was insufficient evidence to support or refute either practice. We aimed to examine the effect of continuous or intermittent rooming-in on breastfeeding duration.

**Methods and findings:**

We included all prospective controlled studies (randomised and non-randomised) comparing rooming-in to nursery care that reported full or partial breastfeeding up to six months. We used the 2016 search results of the Cochrane review and updated the search to August 2018 using OVID MEDLINE. Duplicate data extraction and assessment of risk of bias were performed. Meta-analyses were performed using REVMAN 5. The GRADE approach was used to assess quality of evidence.

Seven studies were included, five had 24-hour-per-day, one daytime only and one 8-hours-per-day rooming-in. Four studies had at least one additional co-intervention: Differences in delivery room management, and educational packages.

All studies contributing to meta-analyses had 24-hour rooming-in. There was no difference in the proportion of infants on full breastfeeding at 3 months (RR 1.14; 95% CI 0.84 to 1.54; very-low-quality evidence), 4 months (RR 0.99; 95% CI 0.73 to 1.33; very-low-quality evidence) and 6 months (RR 0.95; 95% CI 0.57 to 1.58; low-quality evidence). The proportion of infants on partial breastfeeding at 3–4 months was higher with rooming-in (RR 1.31; 95% CI 1.06 to 1.61; very-low-quality evidence).

**Conclusion:**

The addition of non-randomised prospective controlled studies to existing evidence did not add further information on the effects of rooming-in on breastfeeding duration but resulted in lower quality of evidence. Uncertainty about the effects of rooming-in on breastfeeding duration remains.

## Introduction

Breastfeeding is well known for its numerous benefits to both mothers and babies [1]. The World Health Organisation (WHO) recommends a minimum six-month duration of exclusive breastfeeding for newborns [2]. To promote and support breastfeeding, WHO and the United Nations Children’s Fund (UNICEF) developed the Baby-friendly Hospital Initiative (BFHI) [3]. BFHI consists of 10 steps to be practiced by maternity care facilities. Step 7 of these 10 steps is to practice rooming-in, allowing mothers and infants to remain together 24 hours a day. A baby who is not rooming-in with his/her mother would be cared for in a separate nursery, away from the mother from the time of leaving the delivery unit until discharge, during which, the mother may have access to feed her baby; or alternatively her baby may be brought to her for feeding [4]. Rooming-in and nursery care are both traditional practices in many cultures, where they are considered to have their own advantages and disadvantages [5–7].

The recent update of the Cochrane Review, “Rooming-in for new mother and infant versus separate care for increasing the duration of breastfeeding”, (August 2016) [8], found no evidence to support either of the practices for improving breastfeeding outcomes. The authors included randomised control trials (RCTs) comparing 24-hour-per-day rooming-in with nursery care that reported breastfeeding outcomes. Out of the 19 relevant studies that were found, only one study was included. Of the 18 excluded studies, four were excluded because they were not RCTs, four had an intermittent rooming-in intervention [9], (such as daytime rooming-in), two did not have a nursery care group, six investigated the effect of early contact instead of rooming-in, one did not report relevant pre-specified outcomes and one further paper did not have sufficient information to be included or excluded.

The Cochrane authors chose to include only studies with 24-hours-per-day rooming-in thus making assumption that any rooming-in less than 24 hours per day would not provide breastfeeding protection. However, this notion has been queried and has been described as a rigid, oppressive and compulsory hospital routine [10]. For example, maternal difficulty in resting and tiredness has been described as a drawback. If a dose-response effect existed, a shorter duration of rooming-in with intermittent separation, i.e. intermittent or flexible rooming-in, might also be beneficial [10]. In other words, the examination of the evidence for intermittent rooming-in as well as continuous 24-hours-per-day rooming-in during hospital stay might add to the currently available body of evidence on the question of whether rooming-in has any effect on breastfeeding duration.

We postulated that by including non-randomised prospective controlled studies and studies with intermittent rooming-in as well as continuous rooming-in might provide additional information and contribute to the body of knowledge on the effect of rooming-in on breastfeeding outcomes. Therefore, the objective of this systematic review is to examine the effect of rooming-in of any duration (continuous or intermittent) compared with nursery care on breastfeeding outcomes on both primiparas and multiparas by including prospective controlled studies of any design.

## Methods

Apart from the change in the inclusion criteria and breastfeeding definition, we followed the protocol of the Cochrane review [8].

### Inclusion and exclusion criteria

We included all prospective controlled studies examining the effect of any duration of rooming-in (continuous or intermittent) during hospital stay compared with nursery care that reported breastfeeding outcomes.

We excluded studies that only examined rooming-in practices in the delivery room or after hospital discharge, and studies that did not report any breastfeeding outcomes.

### Definition

We defined continuous rooming-in as placing mother and baby in the same room next to each other for 24 hours per day, soon after leaving the delivery room until hospital discharge. Intermittent rooming-in means mothers practiced rooming-in with intermittent separation such as separation at night time. We defined nursery care as placing mother in a postnatal ward while baby was placed in a separate nursery. For nursery care, a mother may have had access to the nursery to feed her baby or alternatively, her baby could be brought to her for feeding.

### Outcomes measurement

We followed the breastfeeding definition of Labbok et al. [11]. Our primary outcomes were the proportion of infants on full breastfeeding (exclusive and almost exclusive), and partial breastfeeding at selected time points such as 3 months, 4 months, and 6 months of age.

Exclusive breastfeeding means that breastmilk and nothing else is given to the baby [11]. Almost exclusive breastfeeding refers to breastfeeding and vitamins, juice, water, minerals, or ritualistic feed given infrequently in addition to breast milk [11]. Partial breastfeeding means the infant is given some breastmilk along with complementary feeding including milk, cereal, or other food or water [11].

We also reported the following breastfeeding related outcomes if they were reported in the included studies: breastfeeding frequency, maternal confidence in breastfeeding, satisfaction in breastfeeding, bonding between mothers and babies, as well as maternal and infant adverse events such as infection in infants, infant crying episodes and maternal wound breakdown, puerperal sepsis, fainting episodes, postpartum haemorrhage.

### Search strategy

The original search was done on the 30th May 2016 by the Cochrane Pregnancy and Childbirth Group. The results of the search are published in the Cochrane review. We obtained the results of the search from the authors of the Cochrane Review. We updated the search by searching only OVID MEDLINE using the search strategy from the Cochrane Pregnancy and Childbirth Group [12]. We limited the search date from 2016 to week 2 August 2018. The detail of the search strategy can be found on the Cochrane pregnancy and childbirth website [12]. Two authors independently went through the titles and abstracts and eliminated those not related. After agreement we obtained the full text articles for the remaining.

### Selection of studies, data extraction, and assessment of risk of bias

We used the standard Cochrane methods for selection of studies, and data extraction [13]. Decisions for inclusion or exclusion were made independently by two authors and any disagreement was resolved through discussion or consultation of the third author. This method was also applied to data extraction. For data extraction, we used a specially designed data extraction form. Where an outcome was presented as a survival curve, we enlarged the figure and a graph paper was overlaid to estimate the survival proportion rate at our pre-specified time points. For studies with more than two arms, we combined groups with a rooming-in intervention into the intervention group, and groups with nursery care practice were combined into the control group, resulting in a single pair-wise comparison. We contacted the author of one study for data.

We assessed risk of bias using the following domains: selection bias (sequence generation and allocation concealment), blinding (study personnel and outcome assessors), attrition bias, reporting bias and other biases. We classified each of the domains as low, high, or unclear risk of bias as described in the Cochrane handbook [14].

Finally, for the primary outcome, we assessed the overall quality of evidence by using GRADE approach developed by GRADE working group [15].

### Analysis

We entered the data into REVMAN 5 software. Dichotomous breastfeeding outcomes were presented as risk ratios (RR) with 95% confidence intervals (CI). Meta-analysis was performed using Mantel-Haenszel fixed effect model.

We tested for statistical heterogeneity in each meta-analysis using the I^2^ and Chi^2^ statistics. We regarded heterogeneity as substantial if I^2^ was greater than 30% or if there was a low P value (less than 0.10) in the Chi^2^ test for heterogeneity. If heterogeneity was found, we attempted to explore the reasons.

In an attempt to explain any heterogeneity we intended to perform a limited number of subgroup analyses and we chose the subgroup analyses stated in the Cochrane Review [8], which were mode of delivery, parity and infant sleeping location. However, we only had sufficient data to perform subgroup analysis by parity.

Wherever possible we analysed outcomes on an intention-to-treat basis.

We planned a sensitivity analysis for the primary outcomes by trial quality (RCT versus NRSs) but we did not do this because there was only one RCT [16] in the meta-analysis.

## Results

**Fig 1:**
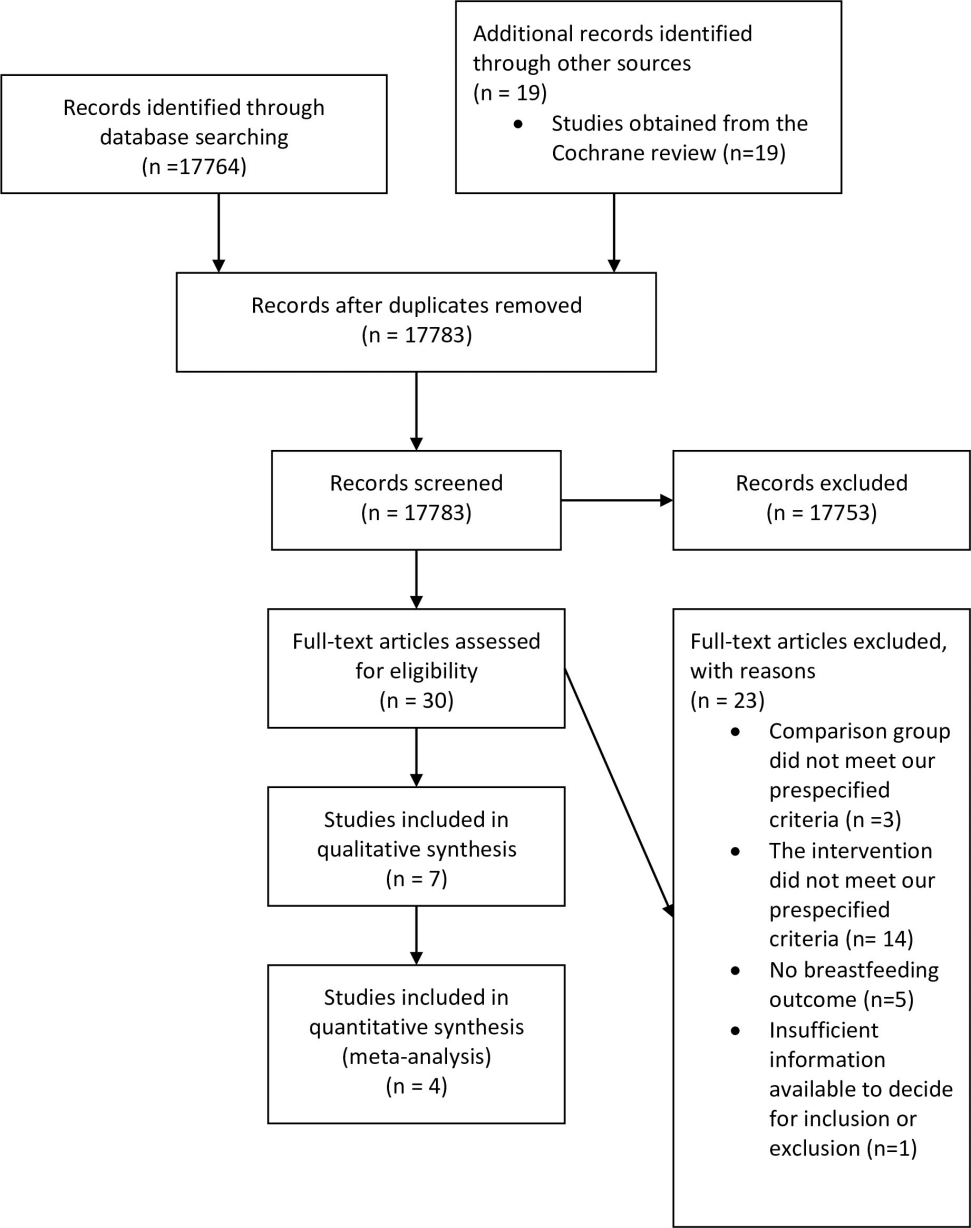
PRISMA flow chart of the included the studies.

Fig 1 is the PRISMA flow chart of the included studies. Our search on OVID MEDLINE yielded 17764 records, of which only eleven articles were relevant, and their full texts were obtained [17–27]. Together with the relevant studies from the search results we obtained from the Cochrane Review authors [8], there were 30 relevant studies [16–45]. Seven [16, 32, 33, 35, 42–44] out of the 30 studies met the inclusion criteria while the other 23 were excluded from this review because of the following reasons: the comparison group was not nursery care as pre-specified [17, 19, 40], the type of room-sharing was compared instead of rooming-in versus nursery care [18, 22, 26, 28, 29], the intervention was not rooming-in but early mother-infant contact versus separation immediately after delivery [30, 37, 41], the intervention was a breastfeeding promotion programme and not rooming-in versus nursery care [20, 24, 25], the interventions was skin to skin contact and not rooming-in [23], there were no breastfeeding outcomes reported [31, 34, 36, 38, 39], they were systematic reviews looking for factors to increase breastfeeding but rooming-in was not studied [21, 27], or there was insufficient information available to make a decision of inclusion or exclusion [45].

### Included studies

Of the seven prospective controlled studies included in this systematic review, two were RCTs [16, 44], five were non-randomised prospective controlled studies [32, 33, 35, 42, 43]. Five of them (one RCT [16], four non-randomised prospective controlled studies [32, 33, 35, 42]) had 24-hour-per-day rooming-in, one RCT had rooming-in during the daytime [44] and one quasi-random study had 8-hour-per-day rooming-in [43]. Breastfeeding duration was reported in six studies [16, 32, 33, 35, 42, 44], four of which had sufficient information to include in the meta-analysis [16, 32, 33, 35] and one reported breastfeeding problems [43]. Of the four studies [16, 32, 33, 35] included in the meta-analysis, three reported full breastfeeding [16, 32, 33] and two reported partial breastfeeding [32, 33]. One further study reported ‘breastfeeding’ [35]. We made an ad hoc decision to include this as partial breastfeeding and tested this judgement with a sensitivity analysis. The timepoints of outcome measurement included 3, 4 and 6 months of age for full (exclusive and almost exclusive), and 3 and 4 months of age for partial breastfeeding. One non-randomised prospective study reported full breastfeeding at ‘up to two months’ [42]. One of the remaining two studies was an RCT which was reported only as an abstract, had a day-time rooming-in intervention, and the outcome was the proportion of infants on exclusive breastfeeding at an undefined time point [44]. The remaining one study was an RCT [43] with an eight-hour-per-day rooming-in intervention, but the duration of breastfeeding or the proportion of infants on full or partial breastfeeding at any of our pre-specified time points was not reported. This study reported outcomes collected by interviewing mothers in the first week after childbirth. Of the outcomes reported, only one outcome, maternal-perceived breastfeeding problems, could be included in the review. Three studies [16, 32, 35] reported the frequency of breastfeeding during the intervention period. Three studies recruited only primiparous women [33, 43, 44], and two studies stratified participants into primiparas and multiparas for analysis [16, 32], for one of these [16], parity data was supplied by the authors. We contacted the authors of Bystrova et al. for further data [16].

Four studies had more than two arms [16, 32, 33, 35]. Bystrova et al. [16] had four arms where we combined the three arms with rooming-in but with different delivery room management. Lindenberg et al. [33] had three groups: continuous post-partum contact (i.e. rooming-in) with standardised breastfeeding promotion, 45 minutes contact in the delivery room followed by complete separation with standardised breastfeeding promotion, and complete separation from birth with ad hoc breastfeeding promotion. We classified the first group as the intervention and combined the latter two groups as nursery care. Perez Escamilla et al. [32] was a three-arm study, which had one group with rooming-in only, one group with rooming-in and breastfeeding guidance, and one group with complete nursery care. We combined the two groups with rooming-in into the intervention group. Elander et al. [35] was a three-arm study where babies with jaundice were recruited and allocated to either a group where nursery care was practiced, or a second group where rooming-in was practised along with two hours instruction on how to care for infants. The third group consisted of healthy babies with no jaundice who were recruited separately and were allocated to rooming-in. We compared the first two groups because we considered the third group to be from a different population. Table 1 summarises the included studies.

**Table 1:** Characteristic of included studies.

Author	Study design	Setting/ study period/ Sample size	Study population, inclusion criteria	Rooming-in intervention	Nursery care intervention	Breastfeeding outcomes/time point
Bystrova et al. [16]	RCT	Maternity home in Russia; 1995–1998 n = 153	Healthy mothers of any parity; uncomplicated pregnancies; normal vaginal deliveries; full term babies	1. Skin-to-skin on mother's chest in delivery room followed by 24-hour rooming-in 2. Dressed and placed in mother's arm for 25–120 minutes in delivery room followed by 24-hour rooming-in 3. Placed in separate cot in delivery room followed by 24-hour rooming-in	Placed in a cot in delivery room followed by nursery care	'Nearly exclusive breastfeeding’ (we defined it as full breastfeeding) at 3, 4, and 6 months, frequency of breastfeeding per day and duration of each feed (minutes) on day 4
Elander et al. [35]	Quasi-RCT Alternating allocation	A hospital in Sweden; 1983–1984 n = 29	Maternal parity not stated Healthy infants who developed jaundice requiring phototherapy; gestational age: 37–41 weeks	24 hours per day, with 2 hours instruction on how to care for infants	Mothers had free access to babies. No additional instruction on how to care for infants	Breastfeeding not defined. Results extrapolated from survival curves. Time points: after discharge and at 3 months (12 weeks)
Greenberg et al. [43]	Quasi random Arm allocation was affected by bed availability	Hospital in Sweden; unknown study date n = 100	Primiparous women; baby >37 weeks or 2500 g, born via vaginal delivery	8 hours per day starting after 12–36 hours of life	Regular feeding schedule with 20 minutes interaction at each feeding	Breastfeeding problems reported by mothers in the first week after infant’s birth
Lind et al. (abstract only) [44]	RCT	Hospital in Sweden; unknown study date n = 344	Primiparous women; uncomplicated pregnancies, deliveries, and puerperium; mothers with enough breastmilk for their babies upon leaving the hospital; baby’s birthweight between 3000 – 4000g	Day-time only	Maternal access not described	Exclusive breastfeeding at unknown timepoint (timepoint was described as first months)
Lindenberg et al. [33]	Before-and-after study	Hospital in Nicaragua; 1982–1983 n = 375	Primiparous women; from poor urban areas of Managua; normal vaginal deliveries	24 hours per day with breastfeeding promotion	Two control groups 1. Complete separation from birth with breastfeeding promotion 2. 45 min mother-infant contact immediately after birth followed by complete separation with breastfeeding promotion	Exclusive and partial breastfeeding at 1 week and 4 months
Perez-Escamilla et al. [32]	Prospective cohort study	2 hospitals in Mexico; 1989 n = 165	Healthy women who planned to breastfeed; mothers of all parity; normal vaginal deliveries; healthy term babies of >2.5kg; Apgar score at 1 and 5 min >7	Two intervention groups 1. 24 hours per day 2. 24-hours per day with breastfeeding guidance	Maternal access not stated	Full (exclusive and almost exclusive), partial and any breastfeeding, plotted as a survival curve from 0 to 4 months
Sousa et al. (abstract only) [42]	Two groups, method of allocation unclear	Maternity ward in Brazil; unknown study date n = 200	Maternal parity not stated; normal full-term babies	24 hours per day	Mothers visit nursery for 30 minutes every 3 hours	Successful (Full) breastfeeding up to 2 months

### Risk of bias

Details of the assessment of risk of bias of the included studies is shown in Table 2. Overall selection bias was judged as high because only two studies were described as randomised [16, 44]. Blinding of participants and personnel to the intervention would have been difficult if not impossible. We judged performance bias to be at low risk if the participants and healthcare personnel were blinded to the study objectives. Three studies reported that mothers were blinded to the purpose of the studies [32, 33, 35], but only one of them stated that the personnel were blinded as well [35]. One study reported blinding of outcome assessors [32] and one reported blinding to the nature of the study [33]. We judged that long-term breastfeeding outcomes were not likely to be affected by lack of blinding of outcome assessor due to the objectivity of the outcome. Risk of attrition bias was unclear or high in five studies [16, 32, 33, 42, 44] and low in two studies [35, 43]. We did not find any risk of selective reporting. We interpreted the presence of additional co-intervention in the intervention group in two studies as high risk of ‘other bias’, [33, 35].

**Table 2:** Assessment of risk of bias.

Studies	Random sequence generation and allocation concealment (selection bias)	Blinding of participants and personnel^2^ and outcome assessors^4^ (performance and detection bias)	Incomplete outcome data (attrition bias)	Selective reporting (reporting bias)	Other bias
Bystrova et al. [16]	Low	High	High	Low	Low
	Table of allocation sequence Sequentially numbered, sealed, opaque envelope used	Not described	Attrition due to treatment refusal in the nursery care group was higher in nursery care than rooming-in (11% vs 4%)	Not detected	Not detected
Elander et al. [35]	High	Low	Low	Low	High
	Alternating allocation but affected by bed availability	Participants and personnel were blinded to the purpose of the study	No attrition	Not detected	The rooming in group also received Infant care guidance which could independently affect outcome
Greenberg et al. [43]^a^	High	High	Low	Low	Low
	Randomised, but allocation was affected by bed availability	No blinding. Outcome self-reported	No attrition	Not detected	Not detected
Lind et al. [44] ^3^ (abstract only)	Unclear	Unclear	Unclear	Unclear	Low
	Randomised. Randomisation and allocation method not described	Not described	Details of attrition not provided	Insufficient information available for judgement	Not detected
Lindenberg et al. [33]	High	Unclear	Unclear	Low	High
	Before (nursery care)-and-after (rooming-in) comparison. Allocation concealment impossible	“Both mothers and interviewers were blind to the purpose of the study.” Blinding of healthcare personnel to the purpose of the study was not described	Overall attrition rate was 27%. Details of attrition rate in individual groups was not reported	Not detected	The rooming in group also received ‘standardised breastfeeding promotion’ which could independently affect outcome
Perez-Escamilla et al. [32]	High	Low	High	Low	Low
	Allocation was by participant preference	Participants were blinded to the study objectives and the existence of different groups. We judged that lack of blinding of the care takers did not influence the outcome because the rooming-in and nursery care groups were in different hospitals	Imbalance of attrition distribution across the groups (2% versus 14% versus 14%). Reasons for attrition not reported	Not detected	Not detected
Sousa et al. [42] ^3^ (abstract only)	Unclear	Unclear	Unclear	Unclear	Low
	Method of allocation not described	Not described	Not described	Not described	Not detected

^1.^ Using Cochrane risk of bias tool [14]

^2^ We judged that blinding of participants and personnel to the intervention was not impossible but that blinding of participants and personnel to the study objectives means low risk of performance bias.

^3^ No full text available. Risk of bias assessment based on the abstract.

^4^ We judged the outcome breastfeeding at 3, 4 and 6 months of age to be an objective outcome not likely to be affected by lack of blinding of the outcome assessor. Hence, six studies that measured these breastfeeding outcomes had low risk of detection bias, the other one study^a^ that measured maternal self-reported breastfeeding problems had high risk of detection bias due to lack of blinding of outcome assessor.

### Effect of rooming-in on full breastfeeding (exclusive or almost exclusive at 3, 4, and 6 months of age)

We combined the outcomes of exclusive and almost exclusive breastfeeding and analysed the proportion of infants on full breastfeeding. Four studies [16, 32, 33, 42] reported this outcome. Bystrova et al. [16] reported proportion of infants on full breastfeeding at 3, 4 and 6 months of age while Lindenberg et al. [33] reported proportion of infants on exclusive breastfeeding at 4 months of age, and Perez-Escamilla et al. [32] reported proportion on full (exclusive and almost exclusive) breastfeeding at four months of age. Lind et al. [44], reported a significant increase in proportion of infants on exclusive breastfeeding among primiparous women in the group with daytime rooming-in compared with nursery care, and Sousa et al. [42] reported a higher proportion of infants on full breastfeeding in the rooming-in group but for both these studies the time-point of outcome measurement was not clear.

#### Proportion of infant on full breastfeeding at 3 months of age

**Fig 2:**
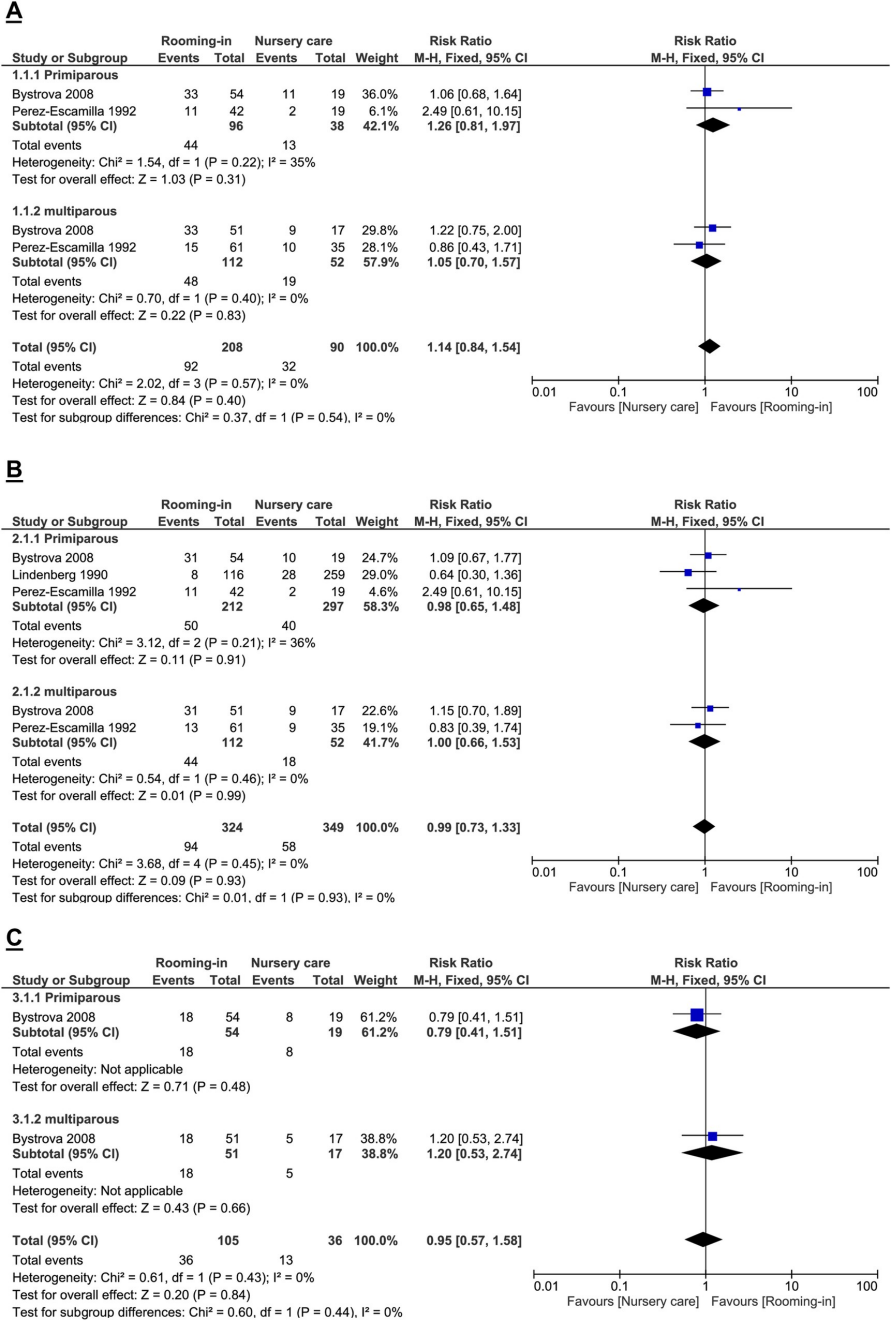
Full breastfeeding. (A) at 3 months, (B) 4 months (C) 6 months of age.

We did not find any difference between rooming-in and nursery care in proportion of infants on full (exclusive and almost exclusive) breastfeeding at three months of age (RR 1.14; 95% CI 0.84 to 1.54; 2 studies, 298 participants; I^2^ 0%; very-low-quality evidence) (Fig 2A). We downgraded the quality two levels for study limitations (one study was non-randomised prospective controlled study and attrition bias) and one level for precision (wide confidence interval which includes harm and appreciable benefit).

#### Proportion of infant on full breastfeeding at 4 months of age

We did not find any difference between rooming-in and nursery care in proportion of infants on full (exclusive or almost exclusive) breastfeeding at four months of age (RR 0.99; 95% CI 0.73 to 1.33; 3 studies, 673 participants; I^2^ 0%; very-low-quality evidence) (Fig 2B). We downgraded the quality two levels for study limitations (two studies were non-randomised prospective controlled study) and one level for precision (wide confidence interval).

#### Proportion of infant on full breastfeeding at 6 months of age

We found no difference in proportion of infants on full breastfeeding at six months of age (RR 0.95; 95% CI 0.57 to 1.58; 1 study, 141 participants; low-quality evidence) (Fig 2C). We downgraded the evidence one level for indirectness (differences in care other than the intervention) and one level for precision (wide confidence interval).

#### Subgroup analysis by parity

For subgroup analysis by parity (primiparous and multiparous), we found no difference between the groups for full breastfeeding at all 3 time points.

### Effect of rooming-in on partial breastfeeding

#### Proportion of infants on partial breastfeeding at 3–4 months of age

Three studies [32, 33, 35], all with 24-hour rooming-in reported this outcome. Lindenberg et al. [33] and Perez-Escamilla et al. [32] measured proportion of infants on partial breastfeeding at 4 months of age, and Elander et al. [35] measured partial breastfeeding at three months of age. We combined the results into a meta-analysis measuring the proportion of infants on partial breastfeeding at 3–4 months of age. There was a small increase in proportion of infants on partial breastfeeding at 3–4 months of age in the rooming-in compared with nursery care group, (RR 1.31; 95% CI 1.06 to 1.61; 3 studies, 561 participants; I^2^ 22%; very-low-quality evidence). We downgraded the quality two levels for very serious study limitations (3 studies were non-randomised prospective controlled studies) and one level for concerns about indirectness (in all three studies there were co-intervention in the rooming-in group or nursery care group).

On subgroup analysis we found no difference between the subgroups by parity (primiparous, multiparous, and parity not stated), RR for primiparous group 1.29, (95% CI 1.04 to 1.61, 2 studies, n = 436) and RR for multiparous group 0.98 (95% CI 0.43 to 2.27, 1 study, n = 96), chi-square test for subgroup differences was 1.75, I^2^ = 0%. One study did not report the parity. On subgroup analysis of primiparous and multiparous (excluding the study with parity not stated), we found no difference between the primiparous group and multiparous group as well, chi-square = 0.39, I^2^ = 0%.

**Fig 3:**
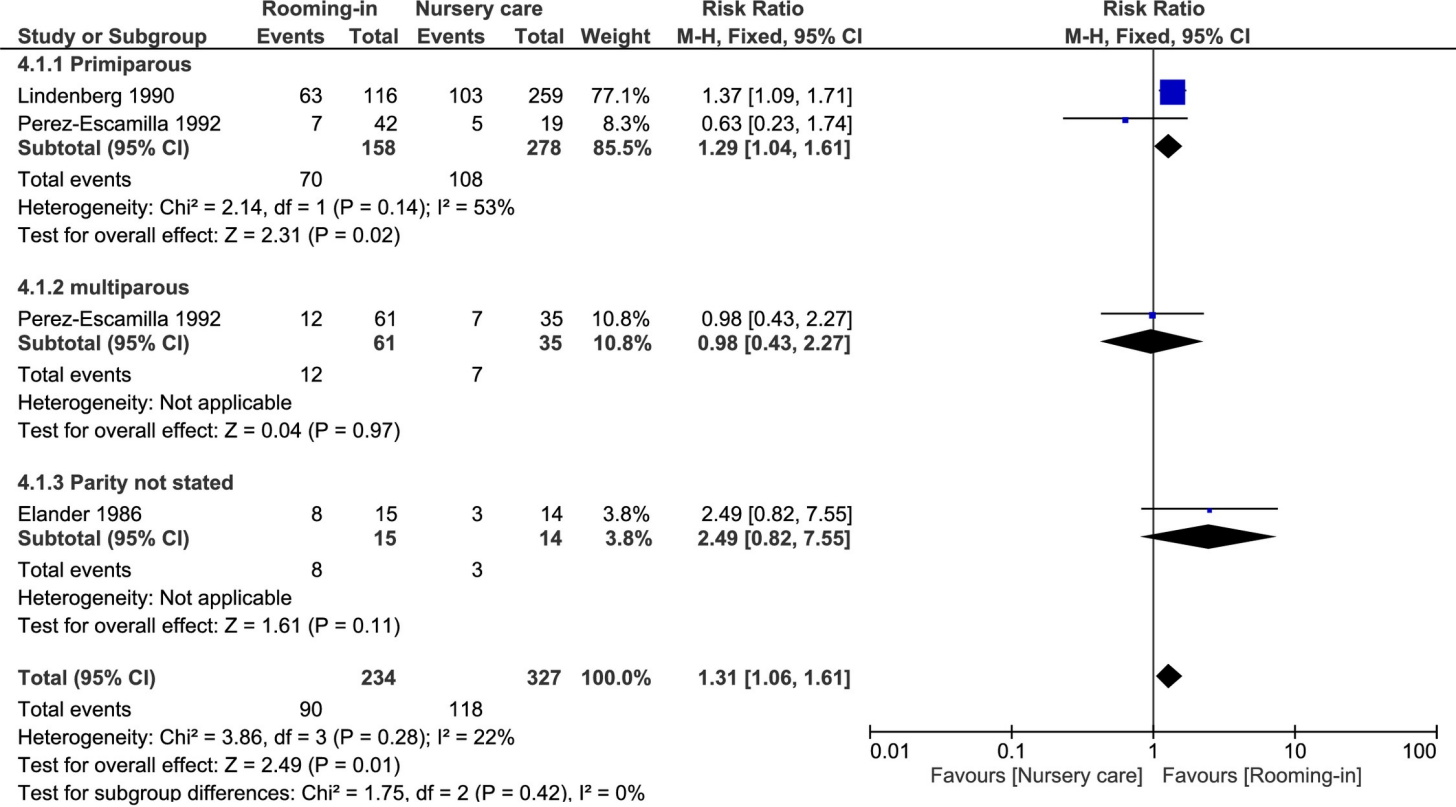
Partial breastfeeding at 3–4 months of age.

Fig 3 showed the forest plot of proportion of infants on partial breastfeeding at 3–4 months of age.

### Other outcomes

Three studies reported frequency of breastfeeds during hospitalisation [16, 32, 35]. Both Bystrova et al. [16] and Perez-Escamilla et al. [32] found an increase in breastfeeding frequency in the rooming-in group. Elander et al. [35] reported an increase in breastfeeding frequency in the rooming-in group but did not report whether this difference was significant. The outcome, frequency of breastfeeds reported in the study by Bystrova et al. could not be included in a meta-analysis because breastfeeding in the nursery care group was scheduled at 7(SD 0) feeds per day for all infants (Table 3).

**Table 3:** Mean breastfeeding frequency (SD) during intervention.

	Rooming-in mean (SD)	Nursery care mean (SD)	P value
Bystrova et al. [16]	8.6 (2.0) n = 109	7 (0) n = 37	Not estimable
Perez-Escamilla et al. [32]	3.7 (2.57) n = 107	0.9 (1.2) n = 58	< .0.001

There was no difference between the groups in self-reported breastfeeding problems in Greenberg et al. [43] (1 study, 100 participants, very-low-quality evidence).

### Sensitivity analysis

Perez Escamilla et al. [32] which measured proportion of infants on full breastfeeding at four months of age had excluded infants who had been given other foods early in life but were exclusively breastfeeding at four months of age. We conducted a sensitivity analysis to assess the effect of re-including these infants. The analysis showed that including these participants did not substantially change the result.

We also did a sensitivity analysis to test our judgement for including the Elander et al. [46] study in the meta-analysis for the outcome partial breastfeeding [35]. This study measured ‘breastfeeding’ at 3 months without specifying whether it was full or partial breastfeeding, while the other two studies in the meta-analysis measured partial breastfeeding at 4 months. There was no substantial change in the effect estimate or 95% confidence interval on exclusion of this study.

## Discussion

We included a total of seven studies on a total of 1366 infants. Overall, we did not find any difference in full breastfeeding at 3, 4 or 6 months except for a small increase in proportion of infants on partial breastfeeding in the rooming-in group at 3–4 months of age. On subgroup analysis although it appeared that this effect was mainly seen in primiparous mothers, the test for subgroup differences did not show any difference between the groups. Therefore, it is not possible to draw any conclusion from this finding. Across all our reported outcomes we judged the evidence to be of very low quality, mostly due to study limitations and precision. The effect sizes for all breastfeeding outcomes were all similar and close to the line of effect. Although there were differences in the study designs there was little statistical heterogeneity for our main outcomes. For four studies there were differences between the two groups other than the intervention. Three studies [32, 33, 35] gave additional breastfeeding education to the rooming-in group and for one study [16] there were differences between the groups in the labour room management. It is possible that this additional education could account for the small difference we found in partial breastfeeding at 3–4 months of ages.

Since the intervention for all the studies contributing to the meta-analysis was 24-hour rooming-in we were not able to demonstrate whether there was a possible dose-response for the period of rooming-in on breastfeeding outcomes.

The reason for including breastfeeding outcomes at three to four months of age was because the current breastfeeding recommendation of exclusive breastfeeding for 6 months is relatively recent [2]. In the past recommendations of 3 and 4 months existed in various settings [46–49] and studies conducted at that time would likely measure these endpoints. We feel it is important that these studies would not be excluded.

Compared with the Cochrane review, by including randomised and non-randomised prospective controlled studies, we increased the study population from 176 participants to over 600 participants for some outcomes. For the two systematic reviews the effect size was similar for the main outcomes with similar precision, but the overall quality of evidence decreased from low quality in the Cochrane review to very low quality in this review.

This illustrates the need for additional high quality randomised controlled trials. However, since 24-hour rooming-in has been included in the WHO BFHI these trials might not now be done unless populations exist that are not yet exposed to BFHI. These two systematic reviews therefore represent the best available evidence on the effect of rooming-in on breastfeeding outcomes. Further studies need to look at the other effects of rooming-in and nursery care. There is little data on the effect of these interventions on breastfeeding problems, maternal confidence or maternal satisfaction.

## Conclusion

In conclusion the addition of evidence from non-randomised prospective controlled studies to that already known from an analysis of RCTs was not able to add any further information on the effects of rooming-in on breastfeeding duration but resulted in lower quality evidence. Uncertainty about the effects of rooming-in on breastfeeding duration remains.

## Supporting information

S1 Checklist PRISMA checklist. (DOC)
